# Quasi-Continuous Metasurface Beam Splitters Enabled by Vector Iterative Fourier Transform Algorithm

**DOI:** 10.3390/ma14041022

**Published:** 2021-02-21

**Authors:** Jinzhe Li, Fei Zhang, Mingbo Pu, Yinghui Guo, Xiong Li, Xiaoliang Ma, Changtao Wang, Xiangang Luo

**Affiliations:** 1 State Key Laboratory of Optical Technologies on Nano-Fabrication and Micro-Engineering, Institute of Optics and Electronics, Chinese Academy of Sciences, P.O. Box 350, Chengdu 610209, China; lijinzhe18@mails.ucas.ac.cn (J.L.); zhangfei@cqu.edu.cn (F.Z.); pmb@ioe.ac.cn (M.P.); gyh@ioe.ac.cn (Y.G.); lixiong@ioe.ac.cn (X.L.); maxl@ioe.ac.cn (X.M.); wangct@ioe.ac.cn (C.W.); 2 School of Optoelectronics, University of Chinese Academy of Sciences, Beijing 100049, China

**Keywords:** quasi-continuous metasurface, beam splitter, iterative Fourier transform algorithm, vector diffraction theory

## Abstract

Quasi-continuous metasurfaces are widely used in various optical systems and their subwavelength structures invalidate traditional design methods based on scalar diffraction theory. Here, a novel vector iterative Fourier transform algorithm (IFTA) is proposed to realize the fast design of quasi-continuous metasurface beam splitters with subwavelength structures. Compared with traditional optimization algorithms that either require extensive numerical simulations or lack accuracy, this method has the advantages of accuracy and low computational cost. As proof-of-concept demonstrations, several beam splitters with custom-tailored diffraction patterns and a 7 × 7 beam splitter are numerically demonstrated, among which the maximal diffraction angle reaches 70° and the best uniformity error reaches 0.0195, showing good consistency with the target energy distribution and these results suggest that the proposed vector IFTA may find wide applications in three-dimensional imaging, lidar techniques, machine vision, and so forth.

## 1. Introduction

The metasurface is an important tool to control light, showing a wide range of potential applications in display [1,2], biomedicine [3,4], illumination source [5], and detection [6]. Especially, asymmetric photonic spin-orbit interactions, enabled by the combination of the geometric phase and propagation phase, have shown great potential to achieve multifunctional devices and systems [7,8]. Different from the traditional metasurface that spatially tailors the geometries of the scatters to introduce the phase discontinuities [9], a quasi-continuous metasurface does not rely on numerous elaborately arranged elements to fulfill various functionalities [10,11,12]. A quasi-continuous metasurface beam splitter divides one laser beam into multiple beams and can be applied in structured light [13,14,15,16], optical interconnects [17], and camera calibration [18,19]. For these applications, the maximal diffraction angle and uniformity error are two important indicators that determine the performance of the device. However, traditional methods based on scalar diffraction theory for designing beam splitters can hardly enable a large diffraction angle and low uniformity error owing to the limitation of paraxial approximation and electromagnetic coupling [20]. When a beam splitter’s maximal diffraction angle becomes larger, its feature size of the structure will be reduced to the wavelength- or even subwavelength-scale, leading to non-ignorable electromagnetic coupling that would cause unpredictable disturbances. Generally, the stronger the coupling is, the larger deviation of actual electromagnetic fields from the target one will be, causing a bigger difference in the diffraction energy distribution between real and theoretical ones.

Due to the reasons mentioned above, vector diffraction theory is necessary for the design of beam splitters with a large diffraction angle. Since vectorial electromagnetic analysis methods are computationally intensive and time-consuming, many efforts have been paid to reduce the simulation time on the premise that the results are accurate enough [21]. However, the major factor that determines the computation load is the times of simulation. For example, famous simulated annealing algorithm and genetic algorithm had been applied to design beam splitters [22,23], but evaluating dozens of samples at each iteration needs lots of computation power and limits these algorithms to achieve the fast design. Adjoint-based optimization method was applied in the design of optical devices recently [21,24,25,26], and beam splitters have also been designed by this method [27]. This method is powerful and flexible but requires twice simulations at each iteration, which increases the time-taking in the optimization process.

In this work, a novel vector iterative Fourier transform algorithm (IFTA) is proposed. Different from the traditional scalar IFTA that imposes constraints in the output spatial frequency domain merely by replacing the amplitude distribution with the target one [28], the proposed method here adjusts the amplitude distribution according to both the target one and results obtained by vectorial electromagnetic simulation. This method can realize the fast design of beam splitters with a wide diffraction angle, since it needs only once vectorial electromagnetic simulation at each iteration and begins to converge at about the 40~60th iteration in the design example of 5 × 5 beam splitters. A 7 × 7 beam splitter with a maximal diffraction angle of 70° is also designed to test the proposed method with different design requirements. These design cases show a good optimization effect and require less computational power compared with the traditional methods mentioned above.

## 2. Materials and Methods

**Figure 1:**
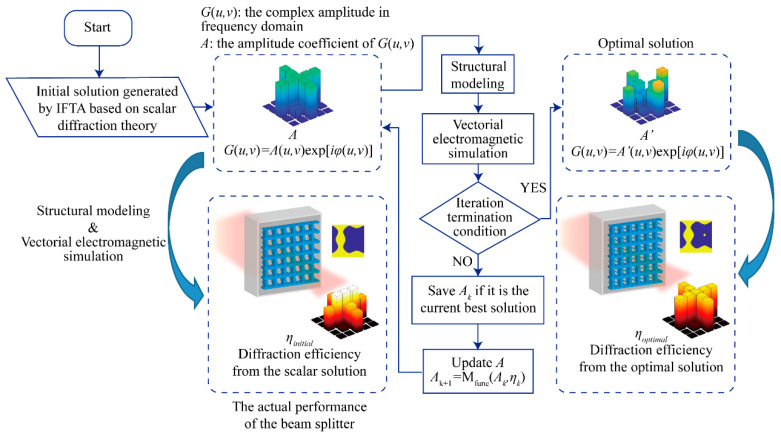
Schematic of the proposed vector IFTA. The flow chart of the optimization algorithm is illustrated combining with the design example of a beam splitter with a cross-shaped diffraction pattern, and each diffraction order is expected to have equal intensity.

Before the whole optimization starts, the period of the beam splitter is determined by the grating equation according to the required diffraction angle and diffraction patterns [29]. After that, to realize a two-level phase distribution, the grating structure height can be determined by:

(1) $$h_{grating}=\frac{\lambda }{2(n_{grating}-1)}$$

where the *n_grating_* is the refractive index of the grating structure, the *λ* is the wavelength of the incident light. With the period and the height of grating structures determined, the grating profile is optimized by the process in Figure 1.

Figure 1 shows the process of the proposed vector IFTA. The optimization needs an initial solution, which can be obtained by the scalar IFTA [28,30], to start the iteration. Beam splitters designed by the scalar IFTA is purely based on scalar diffraction theory, so the corresponding initial solutions are also called scalar solutions. In scalar IFTA, Fourier transformation relates the input plane in the time domain and the output plane in the frequency domain, where constraints are imposed to promote the convergence of the algorithm. The constraints on both planes are about the amplitude. The constraint on the input plane is to replace the amplitude with the amplitude of a plane wave, while on the output plane is to replace the amplitude with the amplitude of the target image. After enough iterations, the output plane in the frequency domain will take on the desired diffraction pattern, and then the corresponding complex amplitude *G*(*u*,*v*) = *A*(*u*,*v*)exp[*iφ*(*u*,*v*)] on the output plane will serve as the initial solution in the proposed vector IFTA, where the *A*(*u*,*v*) means the amplitude distribution, and the *φ*(*u*,*v*) is the phase distribution. The amplitude distribution and the phase distribution are expanded with the aid of the zero-padding approach to enhance the resolution. To get a better initial solution, there is a modification in the scalar IFTA. In practice, it is difficult to obtain the phase distribution, which can well reproduce the target image, merely by the original scalar IFTA. By releasing the constraint in the unconcerned region, significant improvement is found in the image performance on the output plane, and the details about the modification operations as well as the improvement effect are presented in our previous work [31]. The purpose to utilize the modified scalar IFTA to improve the quality of the initial solutions is to exclude their potential adverse effects on the proposed vector algorithm. For instance, if one does not get the target amplitude distribution on the output plane in the scalar simulation, it is hard to tell that if the problem occurs in the initial solution or the algorithm once the optimization result does not meet the demand.

To transform the complex amplitude in the frequency domain into a specific beam splitter model, the first step is to perform inverse Fourier transformation to obtain the complex amplitude in the time domain:

(2) $$g(x,y)=F^{-1}[G(u,v)]$$

The beam splitter is designed to have a binary surface profile, so a binarization operation is imposed on the phase distribution to obtain a new two-level phase distribution:

(3) $$\phi (x,y)=\left\{ \begin{array}{ll} \pi /2 & \;\mathrm{if}\;\arg[g(x,y)]\geq 0\\ -\pi /2 & \;\mathrm{if}\;\arg[g(x,y)]<0 \end{array}\right.$$

where the arg(*) is the function of obtaining phase information. After binarization, the structural material is filled in the design region where ϕ(x,y)=π/2, with the unfilled space being air.

When the period and height of the grating structure is determined, the substrate is properly selected and the grating structure from the initial solution is calculated, the beam splitter’s diffraction efficiency of each order in the *k*th iteration, *η_k_*, can be obtained by performing vectorial electromagnetic simulations. The purpose of the optimization is to achieve an equal intensity of the valid diffraction orders, so the figure of merit (FoM) describing the uniformity of these diffraction orders can be written as uniformity error:

(4) $$\mathrm{FoM}=\frac{\eta_{\max}-\eta_{\min}}{\eta_{\max}+\eta_{\min}}$$

where the *η*_max_ and the *η*_min_ mean the maximal and the minimal diffraction efficiency, respectively. Additionally, they belong to those valid diffraction orders that are expected to exist. For example, the valid diffraction orders in Figure 1 includes nine diffraction orders on the cross pattern. If the FoM is good enough or the number of iteration steps reaches the upper limit, the optimization stops. Otherwise, the *A* with the current best FoM will be saved until the optimization ends if no better solution takes its place in the following iterations, where *A* is updated constantly. The optimal solution is usually obtained on the convergence stage instead of the last iteration step.

The M_func_(*) is the modification function. To realize a uniform energy distribution of each order, the corresponding amplitude coefficient should be modified to suppress the strongest order and enhance the weakest order to promote the uniform energy distribution. Therefore, we choose a modification function given by:

(5) $$A_{\;k+1\_max/min}=M_{\mathrm{func}}(A_{\;k},\eta_{k})=\frac{A_{\;k\_max/min}}{{\left(\eta_{k\_max/min}/\eta_{k\_mean}\right)}^{R}}$$

where the *A_k_max/min_* is the amplitude coefficient corresponds to the diffraction order with the maximal or minimal diffraction efficiency in the *k*th iteration; *η_k___mean_* is the mean value of the *η_k_*; *η_k_max/min_* is the diffraction efficiency of the strongest or the weakest diffraction order; *R* is the disturbance factor to further optimize the results for any possible improvement when the algorithm begins to converge. Note that the *R* varies between 0.7 and 0.9 randomly in the following simulation. The diffraction efficiency of a certain order has a similar change trend with its corresponding amplitude coefficient, and the modification function utilizes this feature to suppress the strongest order and enhance the weakest order by modifying their amplitude coefficients, while the amplitude coefficients correspond to other diffraction orders remain unchanged. Note that the more energy the strongest order has, the stronger the suppression operation will be, which is true of the enhancement of the weakest order.

After optimization, the beam splitter shows a significant improvement in the uniformity of energy distribution, which can be seen by comparing the diffraction efficiency bar charts on the lower left and lower right of Figure 1, respectively.

## 3. Results

### 3.1. The Design of 5 × 5 Beam Splitter with Uniformity Energy Distribution

**Figure 2:**
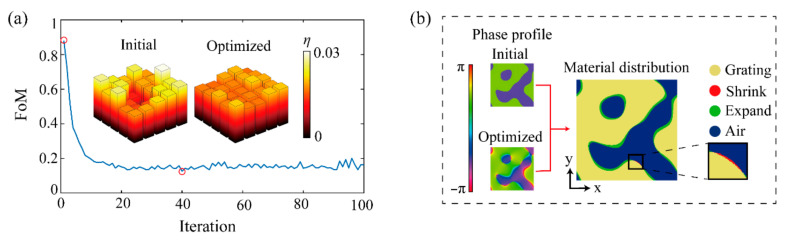
The optimization process of 5 × 5 beam splitter. (**a**) FoM as a function of the iteration in the optimization of the 5 × 5 beam splitter with a maximal diffraction angle of ±35°. The iteration step where the initial solution and the optimal solution are obtained are both marked by the red circles. The bar charts represent the diffraction efficiency of each order from the initial solution and the optimal solution, respectively. (**b**) The phase profiles of the initial and the optimal solution, and the changes in the material distribution from the initial solution to the optimal solution. The red arrow means the operations of structural modeling and comparison. The air, the grating structure and its changes are marked in different colors. The yellow and the blue parts indicate the material distribution remains unchanged after optimization. The green parts mean the grating structure expands to where there was air previously, while the red part, which is magnified to facilitate the observation, indicates the grating structure shrinks after the optimization.

To demonstrate the effectiveness of this method, a simulation model of a beam splitter is constructed with the following parameters: 5 × 5 spot array with the maximal diffraction angle of 35°. Silicon is chosen as the structural material, whose thickness and refractive index is 0.182 μm and 3.60, respectively. Sapphire, with a refractive index of 1.76, is chosen as the material of the substrate. According to the grating equation, the period of the beam splitter’s unit cell is 4.6 μm × 4.6 μm. The incident light has a wavelength of 940 nm and is linearly polarized along the *y*-axis in Figure 2b. The optimization process stops either when the FoM is less than 0.02 or the number of iterations is more than 100. Reticolo, a rigorous coupled wave analysis (RCWA) solver [32], is used to perform the vectorial electromagnetic simulation since RCWA can directly obtain the diffraction efficiency of each order of devices with periodic structures.

Figure 2a shows a design example of a 5 × 5 beam splitter. There is considerable improvement in the FoM from 0.8801 to 0.1264, and the improvement can also be seen by comparing the two bar charts of diffraction efficiency. Furthermore, the relative diffraction efficiency, defined as the ratio of energy needed to the energy transmitted, changes from 89% to 87%. These comparisons above come from the initial solution at the first iteration and the optimal solution at the 40th iteration. Moreover, Figure 2b shows the phase profiles of the initial and the optimal solutions, from which the changes of the material distribution are obtained. It is worth noting that the relative diffraction efficiency drops a little bit after the vector optimization. Though the uniformity error improves significantly, the diffraction efficiency usually drops slightly when the whole vector optimization cycle completes, which does not affect the effectiveness of the proposed algorithm. Because the scalar IFTA has provided the initial solutions with a sufficiently good diffraction efficiency performance while the uniformity error performance is usually unsatisfactory. Therefore, one could hardly promote the two performance indices significantly at the same time if the material of the device remains unchanged. Additionally, the uniformity of energy distribution is more important in practice. That is why the main focus is placed on optimizing the uniformity error of the beam splitter.

### 3.2. The Design of Beam Splitter with Tailored Diffraction Order Pattern

**Figure 3:**
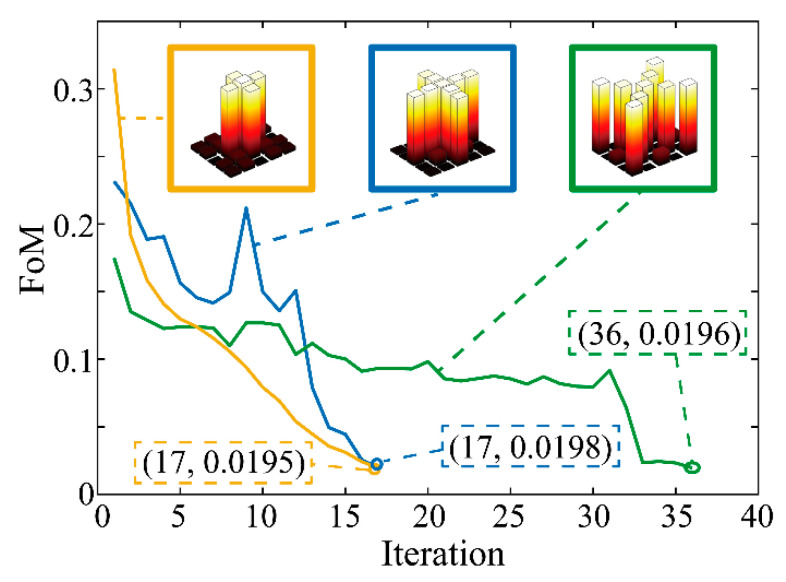
FoM as a function of the iteration in the optimization of beam splitters with tailored diffraction orders. The diffraction order distributions are presented in the upper part of the figure and they are connected with their data curve, whose termination coordinate are also marked. The optimization stops since the termination condition is met.

To demonstrate the applicability of the proposed method, several beam splitters with tailored diffraction orders are designed and the results are recorded in Figure 3. Except for the target diffraction pattern, the other initial parameters are consistent with the 5 × 5 beam splitter’s. The optimization of the three design cases, whose diffraction order distributions are presented from left to right in Figure 3, stops at 17th, 17th, and 36th iteration with an FoM of 0.0195, 0.0198, and 0.0196, respectively. Their relative diffraction efficiencies are 70%, 70% and 67%, respectively. 

## 4. Discussion

As a local optimization algorithm, optimization performance usually varies with different initial solutions. To further analyze their influence, 40 scalar solutions for the 5 × 5 beam splitter are optimized. Among these samples, the best FoM reaches 0.094 while the worst one is 0.390. All the samples have gone through 100 iterations. By checking the number of the vector simulations, one can easily estimate the quantity of the computational cost of an algorithm, since the vector simulation takes enough factors into account to consume a large part of computer’s computational power. As a comparison, Hao et al. [22] designed a beam splitter with the same diffraction angle and spot array by the genetic algorithm, achieving a uniformity error of 0.174. According to the data and description in their paper, the size of a population is 144 and the optimization required about 70 iterations to achieve convergence, so one design requires about 10,080 vector simulations. As shown in Figure 2, the proposed method can achieve better uniformity error using 100 vector simulations, enabling about 100 times more computationally economic. It is worth mentioning that the scalar simulation is not involved in the statistics of computation power consumption, since the scalar simulation costs too little time to make any difference to the total time-taking. For instance, generating one initial solution by scalar IFTA costs 0.29 s, while one vector simulation spends 94.35 s (the time-taking information is obtained from a personal computer with Intel(R) Core(TM) i7-9750H CPU and 16 GB RAM, and it can be further reduced by using a more powerful computer), which means 100 iterations will take about 2.6 h. 

**Figure 4:**
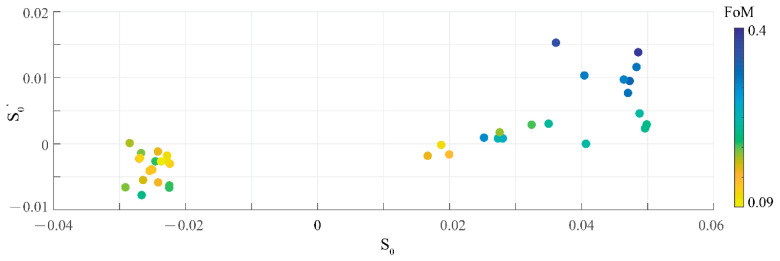
A plot of the relationship of the *S*_0_, the $S_{0}^{\prime }$, and the FoM. The 40 samples are all beam splitters with 5 × 5 spot array and a maximal diffraction angle of 35°. The *S*_0_ is obtained from the initial solutions. The $S_{0}^{\prime }$ and the FoM are obtained from the optimal solutions. All the 40 samples have been optimized for 100 iterations.

After sufficient tests and observation, a phenomenon is found that the (0,0)th order being unable to be suppressed is the main reason for the failure of the optimization. To verify this deduction, a new quantity *S*_0_ is defined in Equation (6) to reveal the relationship between the (0,0)th order and the FoM:

(6) $$S_{0}=\eta (0,0)-\eta_{s}$$

where the *η_s_* is the diffraction efficiency of the second strongest order. Thus, the *S*_0_ shows the extent that the (0,0)th order is stronger than the other orders. The visualization of the data of 40 samples is presented in Figure 4, where the *S*_0_ and the $S_{0}^{\prime }$ are calculated with the data from the initial solution and optimal solution, respectively. In another word, the *S*_0_ and the $S_{0}^{\prime }$ show one characteristics of the device in two different stages: the initial stage (before the vector optimization starts) and the optimal stage. And the deduction that the (0,0)th order is mainly responsible for the failure of the optimization can be confirmed from Figure 4. For those samples with greater *S*_0_ and greater $S_{0}^{\prime }$, their spots tend to have a worse FoM. Another phenomenon found in Figure 4 is that a scalar solution with a weak initial (0,0)th order is more likely to develop into a good one. Moreover, the energy of the (0,0)th order of beam splitters with 0-π binary structures always increases if the deviation of etching depth exists [33]. In another word, if the (0,0)th order is already strong in the simulation, it may tend to be stronger in the experiment, which harms the uniformity of the spot array. Therefore, the control over the (0,0)th order is meaningful both in simulation and practice. Given that the difficulty to suppress the (0,0)th order in vector optimization, the scalar IFTA could provide initial solutions with less energy in the (0,0)th order, since the enhancement is easier than suppression in terms of the (0,0)th order.

**Figure 5:**
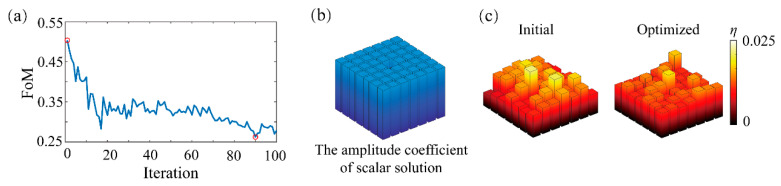
(**a**) FoM as a function of the iteration in the optimization process for the 7 × 7 beam splitter with a maximal diffraction angle of ±70°, which is measured from the (±3, ±3)th order to the (0,0)th order. The iteration where the initial solution and the optimal solution are obtained are marked by red circles. (**b**) The amplitude coefficient of the scalar solution that is used for vector IFTA. It has a suppressed (0,0)th order. (**c**) The comparison of the initial solution’s and the optimal solution’s energy distribution of diffraction orders.

To further demonstrate the applicability of the vector IFTA in a more demanding design case, a beam splitter with a 7 × 7 spot array and maximal diffraction angle of 70° is designed, where the method to control the (0,0)th order is also presented. The unit cell of the device has a period of 4.24 μm, the material of the grating structure and the substrate is titania and silicon dioxide with refractive indices of 2.49 and 1.45, respectively. The thickness of the grating structure is 0.313 μm. The change of the material is a test for the proposed algorithm in a different simulation condition. Given that the failure case and its reason mentioned above, the scalar IFTA provides an initial solution with a suppressed (0,0)th amplitude coefficient, which is illustrated in Figure 5b, while the target of vector optimization remains a beam splitter with a uniform spot array. The optimization achieves the best FoM of 0.2622 at the 90th iteration with 81% relative diffraction efficiency. As can be seen from Figure 5a, it is easy to find that the performance of the 7 × 7 beam splitter is not as good as those of the 5 × 5 beam splitter, which mainly results from the more demanding design requirements instead of the change of material parameters. In another word, the increase of the beams and a larger diffraction angle will make the optimization harder. The improvement is also illustrated by the comparison of the two diffraction efficiency bar charts from the initial solution and the optimal solution in Figure 5c.

## 5. Conclusions

In summary, vector IFTA is used to design beam splitters with a non-paraxial diffraction angle, consuming a very small amount of computational power while achieving a significant improvement in the uniformity of the diffraction energy distribution. An efficient modification function is defined to realize a fast convergence of the algorithm and a detailed search for the optimal solution. Beam splitters with several custom-tailored diffraction order distributions are designed to prove the effectiveness of this method. Among these design cases, the optimization process of the 5 × 5 beam splitter with a maximal diffraction angle of 35° is presented and 40 initial solutions of this kind of beam splitter are optimized to further analyze the relationship between the (0,0)th order and uniformity error, where the best result achieves a uniformity error of 0.094. All three beam splitters with custom-tailored diffraction distributions achieve a uniformity error of less than 0.02. The way to suppress the (0,0)th order is demonstrated in the optimization process of the 7 × 7 beam splitter with a maximal diffraction angle of 70°, and this 7 × 7 beam splitter achieves a uniformity error of 0.2622. This method could promote the applications of beam splitters in different usage scenarios such as depth perception, camera calibration and optical communication.

## Data Availability

The data that support the findings of this study are available within the article.
